# Identification of a circulating microRNAs biomarker panel for non-invasive diagnosis of coronary artery disease: case–control study

**DOI:** 10.1186/s12872-022-02711-9

**Published:** 2022-06-24

**Authors:** Hoda Y. Abdallah, Ranya Hassan, Ahmed Fareed, Mai Abdelgawad, Sally Abdallah Mostafa, Eman Abdel-Moemen Mohammed

**Affiliations:** 1 Medical Genetics Unit, Department of Histology and Cell Biology, Faculty of Medicine, Suez Canal University, Ismailia, 41522 Egypt; 2 Center of Excellence in Molecular & Cellular Medicine, Faculty of Medicine, Suez Canal University, Ismailia, Egypt; 3 Department of Clinical Pathology, Faculty of Medicine, Suez Canal University, Ismailia, 41522 Egypt; 4 Department of Cardiology, Faculty of Medicine, Suez Canal University, Ismailia, 41522 Egypt; 5 Biotechnology and Life Sciences Department, Faculty of Postgraduate Studies for Advanced Sciences (PSAS), Beni-Suef University, Beni-Suef, 62511 Egypt; 6 Department of Medical Biochemistry and Molecular Biology, Faculty of Medicine, Mansoura University, Mansoura, Egypt

**Keywords:** Circulating miRNAs, Coronary artery disease, CAD biomarker, miR-145, miR-182, miR-133a, miR-205

## Abstract

**Background:**

Circulating microRNAs (miRNAs) are considered a hot spot of research that can be employed for monitoring and/or diagnostic purposes in coronary artery disease (CAD). Since different disease features might be reflected on altered profiles or plasma miRNAs concentrations, a combination of miRNAs can provide more reliable non-invasive biomarkers for CAD.

**Subjects and methods:**

We investigated a panel of 14-miRNAs selected using bioinformatics databases and current literature searching for miRNAs involved in CAD using quantitative real-time PCR technique in 73 CAD patients compared to 73 controls followed by function and pathway enrichment analysis for the 14-miRNAs.

**Results:**

Our results revealed three out of the 14 circulating miRNAs understudy; miRNAs miR133a, miR155 and miR208a were downregulated. While 11 miRNAs were up-regulated in a descending order from highest fold change to lowest: miR-182, miR-145, miR-21, miR-126, miR-200b, miR-146A, miR-205, miR-135b, miR-196b, miR-140b and, miR-223. The ROC curve analysis indicated that miR-145, miR-182, miR-133a and, miR-205 were excellent biomarkers with the highest AUCs as biomarkers in CAD. All miRNAs under study except miR-208 revealed a statistically significant relation with dyslipidemia. MiR-126 and miR-155 showed significance with BMI grade, while only miR-133a showed significance with the obese patients in general. MiR-135b and miR-140b showed a significant correlation with the Wall Motion Severity Index. Pathway enrichment analysis for the miRNAS understudy revealed pathways relevant to the fatty acid biosynthesis, ECM-receptor interaction, proteoglycans in cancer, and adherens junction.

**Conclusion:**

The results of this study identified a differentially expressed circulating miRNAs signature that can discriminate CAD patients from normal subjects. These results provide new insights into the significant role of miRNAs expression associated with CAD pathogenesis.

**Supplementary Information:**

The online version contains supplementary material available at 10.1186/s12872-022-02711-9.

## Background

Cardiovascular diseases (CVDs) are one of the top causes of patients’ mortality all over the world [1, 2]. In Egypt, CVDs have also been the leading cause of premature death. Since 1990, they accounted for 46.2% of the overall mortality in Egypt in 2017 [3]. Coronary artery disease (CAD) is the most prevalent among CVDs, and its incidence is high apart of the socioeconomic status of the patient [4]. These figures highlight the urge to discover new CVD biomarkers for the prevention and treatment of those diseases.

Currently, the common diagnosis of CAD is based on coronary angiography, an invasive technique visualizing the positional structure of the coronary artery and it is considered the gold standard for CAD diagnosis [5]. Owing to the known complications of invasive techniques, the emergence of non-invasive and non-imaging techniques offers excellent opportunities [6].

MicroRNAs (miRNAs) are non-coding, single-stranded RNAs with 20–22 nucleotides in length [7]. Their primary function is to block mRNA translation to protein via binding to complementary sequences on messenger RNA (mRNA). About 1,900 unique human miRNAs have been identified till now, and most of them inhibit and target gene expression for hundreds of genes [8]. In addition, it is estimated that miRNAs regulate about 60% of human protein-coding genes and each miRNA targets multiple mRNAs [9].

In the dilemma of discovering non-invasive biomarkers in CVDSs, major scientific endeavors have been turned to the identification of circulating miRNAs as diagnostic, prognostic, and therapeutic biomarkers in many diseases, including CAD [10].

Although several biological molecules, including peptides, proteins, cytokines, and different metabolites, are currently being used as biomarkers for CVDs [11], circulating miRNAs possess many attractive features of biomarkers owing to their stability as they are not degraded by endogenous RNases in the circulation [12, 13].

Several studies have described the role of circulating miRNAs as early diagnostics biomarkers in CAD. At the same time, others demonstrated their prognostic and therapeutic potential interventions in CAD [14]. So, circulating miRNAs are considered now a hot spot of research that can be employed for monitoring and/or diagnostic purposes of CVDs. Moreover, since different disease features might be reflected on altered profiles or plasma/serum miRNAs concentrations, a combination of miRNAs will provide more reliable biomarkers [15].

In this vicinity, our study investigated the differential expression of a panel of 14-miRNAs selected using bioinformatics databases and current literature searching for miRNAs suspected to be involved in CAD pathogenesis and have putative binding sites for the most affected genes in CAD.

## Subjects and methods

### Study population

This study was a case–control study with 146 participants classified into two groups. The first group included 73 patients presenting with symptoms or findings suggesting CAD by clinical examination and diagnostic tools (Echo and ECG) recruited from the cardiology clinic at the Suez Canal University Hospital (SCUH) from June 2020 till June 2021. All details on study subjects are available in Additional files 1, 2.

### Selection of miRNAs under study using bioinformatics tools

The miRNAs under study were selected using bioinformatics online tools as HMDD (http://www.cuilab.cn/) [16], and miR2Disease (http://www.mir2disease.org/) [17]. Also, we searched available literature for the most common miRNAs involved in CAD pathogenesis. All details related to miRNAs selected based on literature are available in Additional file 2.

### Blood samples collection

Three ml of fresh venous blood was collected from all study participants in vacutainer tubes containing ethylene diamine tetraacetic acid (EDTA) anticoagulant. The samples were centrifuged to separate plasma; 100 μl plasma was preserved in 500 μl Qiazole reagent. The plasma samples were stored at − 80℃ till further analysis.

### MicroRNA extraction and quality analysis

Total RNA was isolated using Qiagen miRNeasy Mini kit (cat no 217004, QIAGEN, Hilden, Germany) following the modified protocol supplied by the manufacturer. RNA concentration and purity were determined using NanoDrop 2000 1C spectrophotometer (NanoDrop Tech., Inc. Wilmington, DE, USA).

### Circulating miRNAs relative expression analysis using quantitative real-time PCR assay

The expression profile of 14 circulating miRNAs involved in CAD pathogenesis was assessed in the plasma of all study participants using Real Time-Polymerase Chain Reaction (RT-PCR). This was done via a two-step approach as follows; (a) reverse transcription (RT), and (b) quantitative Real-Time PCR, where the premix of cDNA was used as a template for relative quantification of the 14 human miRNAs under study, which are miR-21-3p, miR-126-5p, miR-145-5p, miR-155-3p, miR-208a-5p, miR-140-3p, miR-182-5p, miR-146a-5p, miR-223-5p, miR-196b-5p, miR-200b-3p, miR-205-5p, miR-133a-5p, and miR-135b-5p. All details related to RT and Real-Time PCR conditions are available in Additional file 2.

### Assessment of circulating miRNAs predictive significance as biomarkers

The contribution to the predictive capacity of the significant miRNAs was analyzed using Receiver Operating Characteristic (ROC) curves to evaluate the diagnostic value of the used miRNAs as biomarkers for CAD pathogenesis. A *p*-value of < 0.05 was considered statistically significant.

### Function and pathway enrichment analysis

The functional enrichment analysis was conducted using the software Database for Annotation Visualization and Integrated Discovery (DAVID) (https://david.ncifcrf.gov/) [18], where gene ontology (GO) consisting of biological processes, cellular components, and molecular functions terms was searched for via Pathway analysis on the Kyoto Encyclopedia of Genes and Genomes (KEGG) database [108] for determining the pathways affected with differential miRNA expression and their target genes. More details on function and pathway enrichment analysis are available in Additional file 2.

### MiRNA‐mRNA regulatory network construction

The targets of the homogenously statistically significant DEmiRNAs were predicted using miRTargetLink 2.0 (Version 2.0, https://ccb-compute.cs.uni-saarland.de/) [19]. More details on miRNA-mRNA regulatory network construction are available in Additional file 2.

### Statistical analysis

Data were analyzed using R software version 3.3.2, GraphPad prism 7, SPSS software version 23.0, and PC-ORD ver. 5.0. We used the G*Power 3.1.9.2. with the specified study design (gene expression), alpha error = 0.05, an effect size = 0.74, and a total sample size of 146 was calculated that can give 80% power of the study http://www.gpower.hhu.de/A [20]. Fold change of the miRNAs was estimated using the LIVAC method (= 2−ΔΔCq) [21]. More details on statistical analysis are available in Additional file 2.

## Results

### Baseline characteristics and CAD risk factors among the study participants

Baseline data from all study participants in both control and CAD groups were presented in table (1). The age of participants showed an average of 38.34 ± 11.90 and 54.93 ± 9.56 years in controls and study groups, respectively. The CAD group showed significantly higher age (*p* < 0.001***) than the control group. Subjects aged over 55 years were nearly five times prone to develop CAD (OR = 4.9, 95% CI: 2.2–10.8, *p* = 0.001) compared to subjects aged 18 to 55 years. The male gender was more represented in control and CAD groups with 52 (71.2%) and 55 (75.3%) patients in the control and the CAD group, respectively, with a non-statistical difference (*p* > 0.05) among the two groups. About CAD risk factors, smokers were significantly (*p* < 0.001***) higher in the CAD group with a total of 42 (57.5%) smokers compared to the control group, which included 21 (28.8%) smokers. Smokers were nearly three times more prone to develop CAD (OR = 3.4, 95% CI: 1.7–6.7, *p* = 0.001) than non-smokers. Family history was found for 45 (61.6%) patients compared to 25 (34.2%) subjects in the control group, with a highly significant difference between the two groups. CAD patients with positive family history were nearly three times more prone to develop CAD (OR = 3.1, 95%CI: 1.6–6.1, p = 0.001) than patients with negative family history. Concerning dyslipidemia, patients were significantly higher in CAD group 59 (80.8%) compared to the control group 3 (4.1%). Dyslipidemia was shown to be a significant risk factor in our CAD patients with nearly 98 times prone to develop CAD (OR = 98.3, 95%CI: 27–358.7, p = 0.001) compared to subjects with standard lipid profile. The average (± SD) BMI of the CAD group (30.16 ± 5.68) was significantly higher (*p* = 0.011) than the control group (27.69 ± 3.99), as shown in Table 1. CAD patients with obesity were nearly two times more to develop CAD (OR = 2.4, 95%CI: 1.2–4.8, p = 0.011) than non-obese patients.

**Table 1:** Baseline characteristics and CAD risk factors among the study participants

Variable	Controls	CAD cases	*P*-value	Odds ratio (95% CI)
Age^t^	38.34 ± 11.90	54.93 ± 9.56	< 0.001***	–
*Age group*^*M*^				
> 55 years	11 (15.1%)	34 (46.6%)	< 0.001***	Reference
< 55 years	62 (84.9%)	39 (53.4%)		4.9 (2.2–10.8)
*Gender*^*M*^				
Males	52 (71.2%)	55 (75.3%)	> 0.05 (ns)	Reference
Females	21 (28.8%)	18 (24.7%)		1.2 (0.6–2.6)
*Smoking*^*M*^				
Smoker	21 (28.8%)	42 (57.5%)	< 0.001***	Reference
Non-smoker	52 (71.2%)	31 (42.5%)		3.4 (1.7–6.7)
*Family history*^*M*^				
Positive	25 (34.2%)	45 (61.6%)	< 0.001***	Reference
Negative	48 (65.8%)	28 (38.4%)		3.1 (1.6–6.1)
*Dyslipidemia*^*M*^				
Dyslipidemia	3 (4.1%)	59 (80.8%)	< 0.001***	Reference
No dyslipidemia	70 (95.9%)	14 (19.2%)		98.3 (27–358.7)
*Obesity*^*M*^				
Obese	21 (28.8%)	36 (49.3%)	0.011*	Reference
Non-obese	52 (71.2%)	37 (50.7%)		2.4 (1.2–4.8)
BMI^t^	27.69 ± 3.99	30.16 ± 5.68	0.003**	–

^*^, **, *** Significant at *p* < 0.05, < 0.01, < 0.001; ns, nonsignificant at *p* > 0.05

^**t**^independent t-test between study and control groups (parametric)

^**M**^Mann–Whitney test between study and control groups (non-parametric)

### Comorbidities, clinical and cardiovascular findings among CAD patients

Table 2 shows the comorbidities clinical and cardiovascular findings among the CAD patients under study. Concerning comorbidities, there was no statistical significance between CAD and either diabetes, hypertension, or ischemic heart disease (IHD) among our study population. Clinical examination revealed an average (± SD) for Body surface area (BSA) of 1.84 ± 0.18, Systole 127.33 ± 17.28, diastole of 81.37 ± 16.14, Left Ventricular Ejection Fraction (LVEF) of 46.58 ± 13.25. Grades of LVEF represented by grades from normal to severe were 22 (30.14%), 15 (20.55%), 24 (32.88%), and 12 (16.44%), respectively. Grade 3 was the highest with a statistically significant difference, as revealed by the Chi-squared test. The average (± SD) WMSI was recorded as 1.49 ± 0.44. The diastolic grade represented from normal to severe were represented by 4 (5.48%), 42 (57.53%), 22 (30.14%), and 5 (6.85%), with a highly significant difference between grades.

**Table 2:** Co-morbidities, clinical and cardiovascular findings among CAD Group represented as frequency (n, %)

Variable	CAD Cases	*P*-value
*Diabetes*		
Diabetics	33 (45.2%)	> 0.05 ns
Non-diabetics	40 (54.8%)	
*Hypertension*		
Hypertensive	32 (43.8%)	> 0.05 ns
Non-hypertensive	41 (56.2%)	
*Ischemic heart disease*		
Ischemic heart disease	41 (56.2%)	> 0.05 ns
Non-ischemic heart disease	31 (42.5%)	
BSA	1.84 ± 0.18	
Systole	127.33 ± 17.28	
Diastole	81.37 ± 16.14	
LVEF	46.58 ± 13.25	
*LVEF grade*		
Normal	22 (30.14%)	< 0.001***
Mild	15 (20.55%)	
Moderate	24 (32.88%)	
Severe	12 (16.44%)	
WMSI	1.49 ± 0.44	
*Diastolic function*		
Normal	4 (5.5%)	< 0.001***
Mild	42 (57.5%)	
Moderate	22 (30.1%)	
Severe	5 (6.8%)	

^***^Significant at *p* < 0.001; ns, nonsignificant at *p* > 0.05 using Chi-square test

### Circulating miRNAs relative expression analysis

**Fig. 1:**
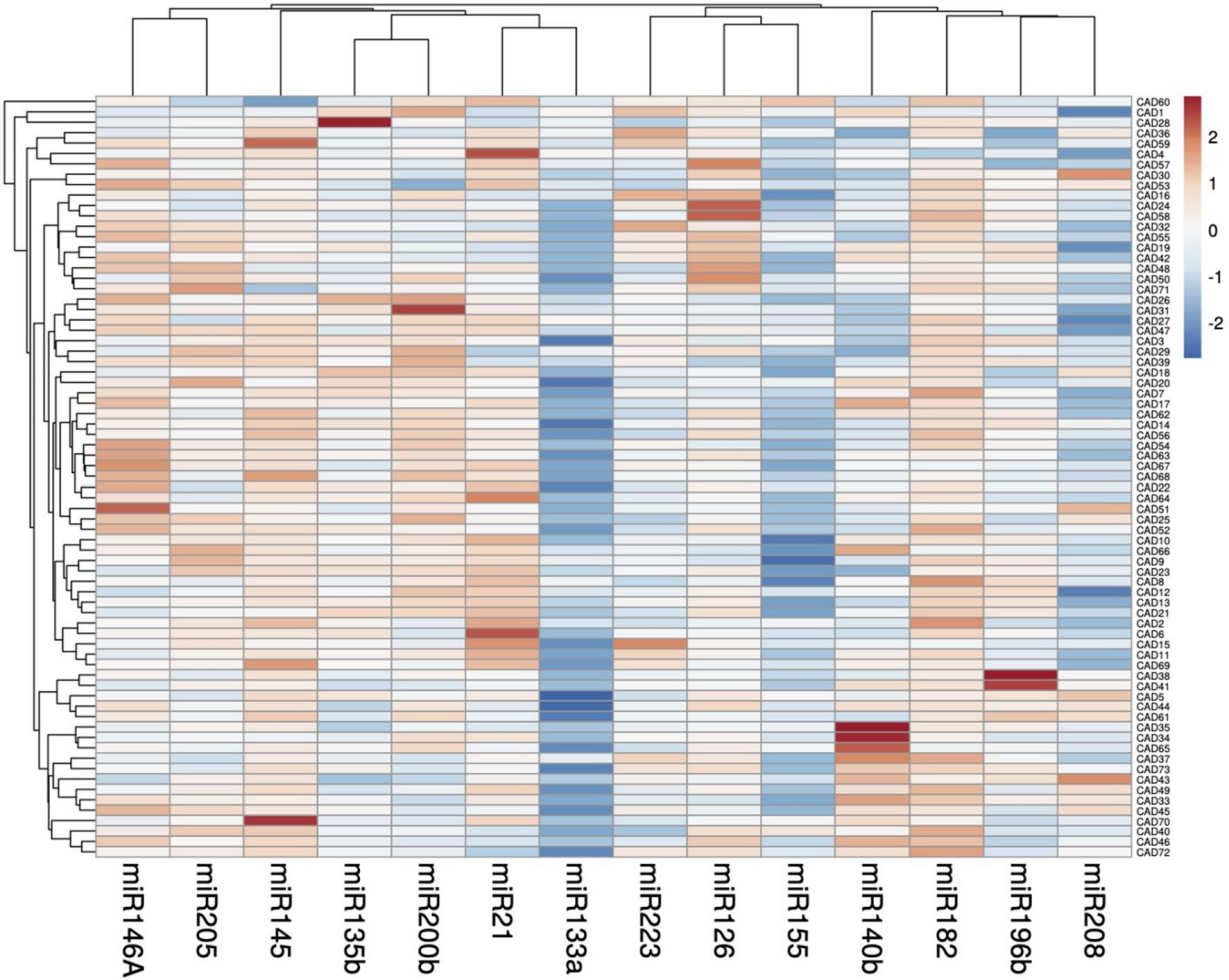
The differential expression profile of circulating miRNAs under study in CAD (n = 73). Heat map illustrates the levels of all miRNAs under study (Log2fold change) in CAD patients. Color grades is shown within each row, with the highest expression corresponding to deep red and the lowest to deep blue

**Fig. 2:**
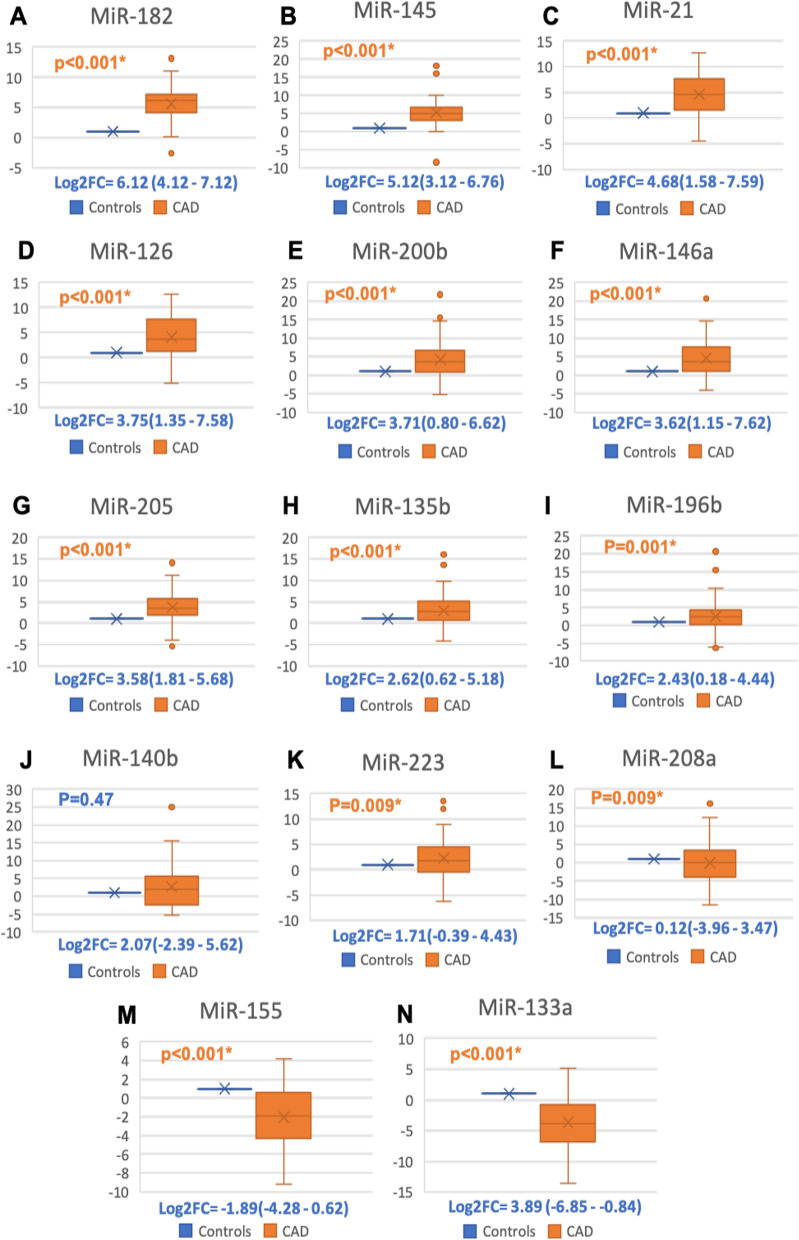
The relative expression level of the circulating miRNAs under study in CAD. Fourteen miRNAs were analyzed: miR-21, miR-126, miR-133a, miR-135b, miR-140, miR-145, miR-146a, miR-155, miR-182, miR-196b, miR-200b, miR-205, miR-208a and miR-223. SNOR68 and RNU6B were used as an endogenous control. The values are represented as median (Q1 and Q3) using Whiskers and bars. All values were log-transformed with the control level sets at the Fold change equals 1. Mann–Whitney U test was used for comparison. *p-Values < 0.05 were considered statistically significant

The differential expression patterns of the 14 miRNAs under study (miR-21, miR-126, miR-133a, miR-135b, miR-140, MiR-145, miR-146a, miR-155, miR-182, miR-196b, miR-200b, miR-205, miR-208a, miR-223) were investigated by qRT‐PCR and shown in (Fig. 1). Out of the 14 circulating miRNAs; miRNAs miR133a, miR155 and miR208a were down-regulated in CAD patients compared to the control group and recorded a median (IQR) of 3.89 (− 6.85 to − 0.84), − 1.89(− 4.28 to − 0.62) and 0.12(− 3.96–3.47) respectively (Fig. 2).

However, when sorting the relative expression patterns in the rest of the up-regulated 11 miRNAs in a descending order from highest fold change to lowest, the following order was obtained: miR-182 6.12 (4.12–7.12), miR-145 5.12(3.12–6.76); miR-21 4.68(1.58–7.59); miR-126 3.75(1.35–7.58), miR-200b 3.71(0.80–6.62), miR-146A 3.62(1.15–7.62), miR-205 3.58(1.81–5.68), miR-135b 2.62(0.62–5.18); miR-196b 2.43(0.18–4.44), miR-140b 2.07(− 2.39–5.62) and, miR-223 1.71(− 0.39–4.43). Differences were assessed by Mann–Whitney where, all miRNAs showed a highly significant difference between study and control groups, except miR-140b showed a non-significant difference (Fig. 2).

### Circulating miRNAs predictive significance as biomarkers by ROC analysis

Receiver operating curve (ROC) including Area Under Curve (AUC) and probability levels were presented in Table 3. The ROC curve data from Table 3 indicated that miR-145, miR-182, miR-133a, miR-205, miR-21, miR-155, miR-126, miR-146A, miR-200b, miR-135b revealed a highly significant (*p* < 0.001***) and valuable biomarkers with the highest AUCs of 0.959, 0.959, 0.863, 0.836, 0.767, 0.767, 0.767, 0.767, 0.740, and 0.712 respectively.

**Table 3:** ROC analysis for biomarker accuracy testing of circulating miRNAs under study

miRNA	AUC				
	Area	SE^a^	Asymptotic Sig.^b^	Asymptotic 95% CI	
				Lower	Upper
miR-145	0.959	0.023	< 0.001***	0.913	1.000
miR-182	0.959	0.023	< 0.001***	0.913	1.000
miR-205	0.836	0.043	< 0.001***	0.751	0.921
miR-133a	0.863	0.040	< 0.001***	0.784	0.942
miR-155	0.767	0.049	< 0.001***	0.670	0.864
miR-126	0.767	0.049	< 0.001***	0.670	0.864
miR-146A	0.767	0.049	< 0.001***	0.670	0.864
miR-21	0.767	0.049	< 0.001***	0.670	0.864
miR-200b	0.740	0.051	< 0.001***	0.639	0.840
miR-135b	0.712	0.053	< 0.001***	0.608	0.816
miR-196b	0.644	0.056	0.003**	0.534	0.754
miR-223	0.616	0.057	0.015*	0.505	0.728
miR-140b	0.589	0.058	0.063 ns	0.476	0.702
miR-208	0.616	0.057	0.015*	0.505	0.728

AUC: 0.5 or less = no discrimination, 0.7–0.8 = acceptable discrimination, 0.8–0.9 = excellent discrimination, and more than 0.9 = outstanding discrimination

Significant *P*-values are in bold

Abbreviations: *AUC* Area under the curve, *SE* Standard error

### Correlation analysis of circulating miRNAs differential expression levels and the CAD patients’ clinical characteristics

**Fig. 3:**
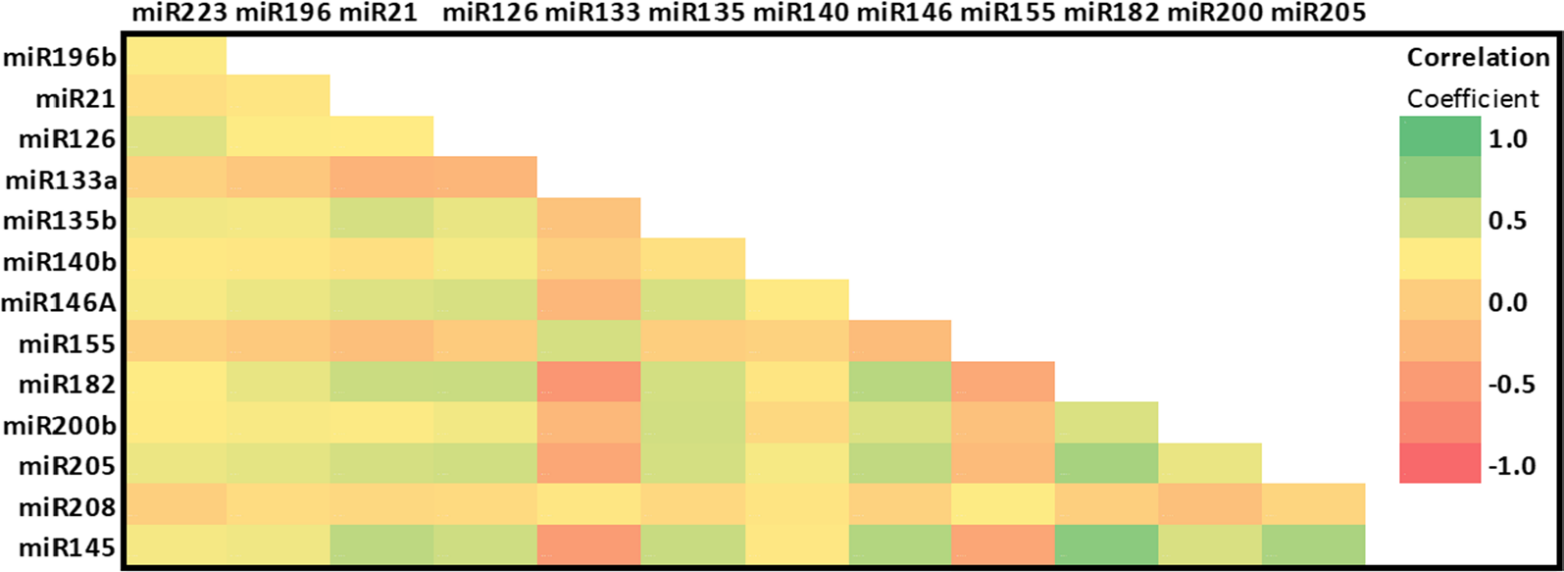
Correlation Matrix between all circulating miRNAs under study

The 14 selected circulating miRNAs showed various distribution among all CAD patients. The Spearman’s rank correlation of the 14 selected plasma miRNAs in both control and CAD was evaluated and presented in Fig. 3. There were a strong association between some of the miRNAs understudy in CAD patients with Spearman’s correlation coefficient of 0.59 and more and a two-tailed significance *p* < 0.0001 (miR-182 and miR-145: r = 0.820***; miR-182 and miR-205: r = 0.693***; miR-145 and miR-205: r = 0.678***; miR-146a vs. miR-182: r = 0.619***; miR-146a vs. miR-145: r = 0.639***; miR-21 vs miR-145: r = 0.595***).

Considering the relation between miRNAs and the clinical data shown in Table 4, most of the studied miRNAs showed positive statistically significant relation with age except miR-140b, miR-196b, and miR-223. MiR-21, miR-126, miR-135b, miR-155, and miR-182 significantly linked with smoking. MiR-133a and miR-182 showed significant association with family history. All miRNAs under study except miR-208 revealed a statistically significant relation with dyslipidemia. MiR-126 and miR-155 showed significance with BMI grade, while only miR-133a showed significance with the obese patients. MiR-140b, miR-182, miR-196b, and miR-208 revealed positive statistically significant relation with hypertension, while miR-21 and miR-145 showed significance with ischemic heart disease. Finally, miR-135b and miR-140b showed a significant correlation concerning the Wall Motion Severity Index.

**Table 4:**
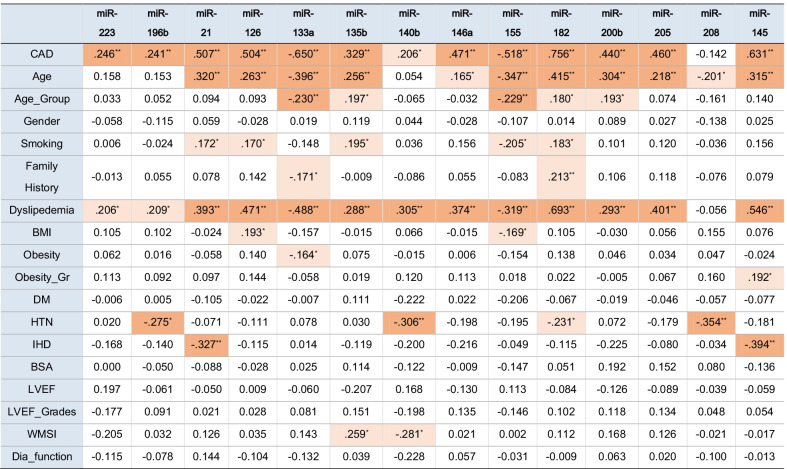
Correlation analysis of circulating miRNAs relative expression levels and the CAD patients’ clinical characteristics

Association of gene expression with clinical features. Pearson’s Correlation coefficient are presented. Significant values are highlighted.

Abbreviations: *CAD* Coronary artery disease, *BMI* Body mass index, *DM* Diabetes mellitus, *HTN* Hypertension, *IHD* Ischemic heart disease, *LVEF* Left ventricular ejection fraction, *WMSI* Wall motion severity index, Dia_Function Diastolic Function

**Correlation is significant at the 0.01 level (2-tailed). *Correlation is significant at the 0.05 level (2-tailed).

### Function and pathway enrichment analysis of circulating miRNAs DE in CAD

**Fig. 4:**
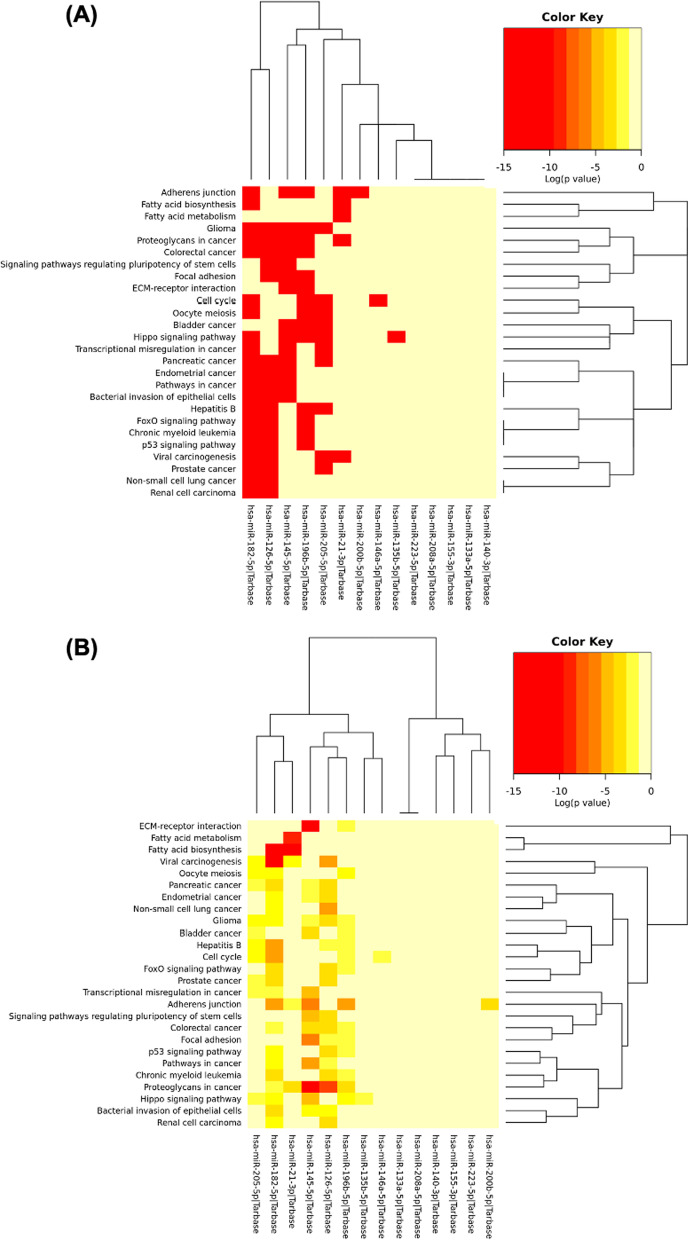
KEGG pathways enriched analysis for differentially expressed circulating miRNAs under study in CAD **A** Using targeted pathways clusters/heatmap. **B** Using significance clusters/heatmap

For identifying all the pathways targeted by DE circulating miRNAs in CAD, a pathway enrichment analysis based on annotated gene targets in GO was performed. The databases were used to assess the 14 miRNAs under study regulatory functions and for identifying the molecular pathways for the miRNAs under study. We used the KEGG pathway database to perform the functional pathway analysis. Enrichment of specific pathways revealed pathways relevant to the fatty acid biosynthesis, ECM-receptor interaction, proteoglycans in cancer, and adherens junction were found as shown in Fig. 4. The fatty acid biosynthesis and ECM-receptor interaction pathways were significantly enriched in CAD patients (Fig. 4B).

**Fig. 5:**
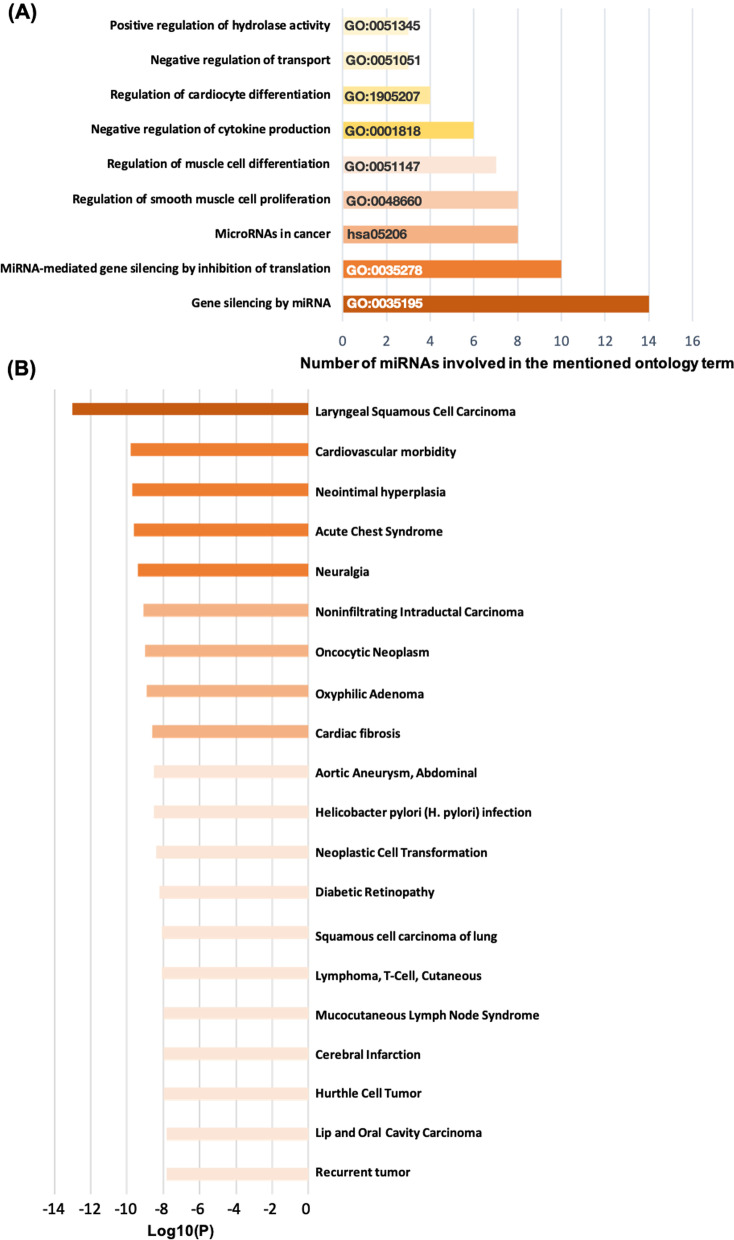
Pathway, process enrichment and association analysis for differentially expressed circulating miRNAs under study in CAD **A** Top 9 clusters with their representative enriched terms (one per cluster) **B** Circulating miRNAs under study enrichment in relation to ontology categories: DisGeNET

The GO biological processes related to CAD pathogenesis were found to be distinctly enriched in our analysis as the enriched pathways were associated with negative regulation of transport, regulation of cardiomyocyte differentiation, negative regulation of cytokine production, regulation of muscle cell differentiation, regulation of smooth muscle cell proliferation, miRNA-mediated gene silencing by inhibition of translation and gene silencing by miRNA as represented in Fig. 5A.

To assure quality control and investigate association analysis, the circulating miRNAs understudy was enriched using DisGeNET, collected and grouped into clusters as shown in Fig. 5B based on the top enriched clusters and their membership similarities where it identified cardiovascular morbidity as one of the top clusters among which our circulating miRNAs are involved.

### MiRNA‐mRNA regulatory network construction

**Fig. 6:**
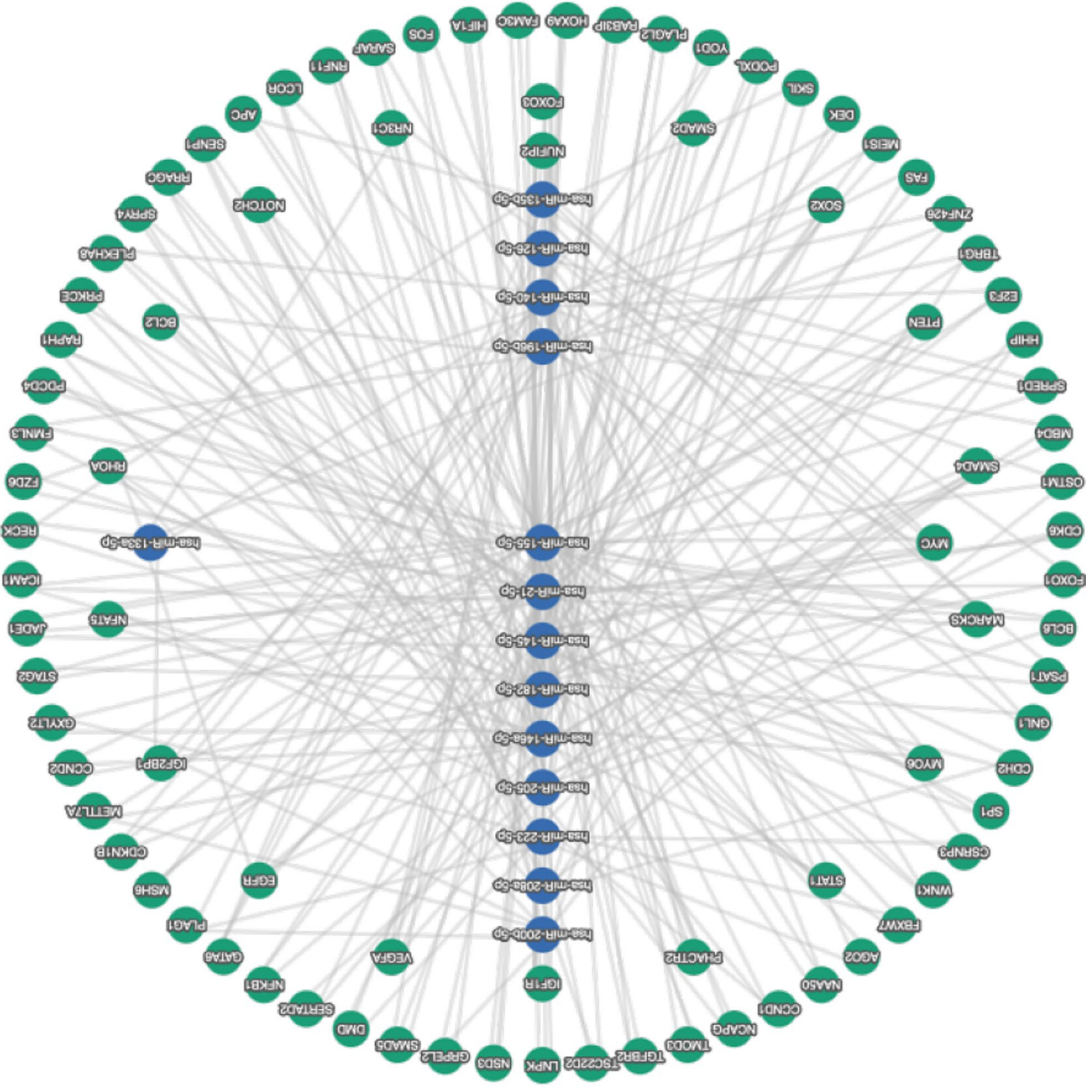
The 14 miRNAs under study target genes network. MiRNA targets were only the validated miRNAs whether with strong or weak validity with additional filter of minimum 3 shared targets using miRTargetLink 2.0 (https://ccb-web.cs.uni-saarland.de/mirtargetlink/network.php)

Our network analysis identified the relationship between the circulating miRNAs under study and their target genes. Our miRNA-target gene network comprised 14 microRNAs and 295 target genes then filtered with a minimum of 3 shared targets that revealed a final of 87 target genes using miRTargetLink 2.0 (https://ccb-web.cs.uni-saarland.de/mirtargetlink/network.php) (Fig. 6). The circulating miRNAs understudy and their targeted genes were related to the biological processes known to be involved in CAD pathogenesis, such as fatty acid biosynthesis, ECM-receptor interaction, proteoglycans in cancer and adherens junction, negative regulation of transport, regulation of cardiomyocyte differentiation, negative regulation of cytokine production, regulation of muscle cell differentiation, regulation of smooth muscle cell proliferation, miRNA-mediated gene silencing by inhibition of translation and gene silencing by miRNA.

Among the critical genes involved in CAD were the SMAD genes that are targeted by six of our circulating miRNAs (miR-135b, miR145, miR146a, miR-155, miR182, and miR-205) for proteins involved in ECM remodeling, cell differentiation, endocardial and epicardial EMT, neural crest migration, and maintenance of cardiovascular structure and function.

Finally, target genes regulated by the circulating miRNAs understudy were also correlated with the FOXO signaling pathway, such as FOXO1 (miR-21 and miR-135b), FOXO3 (miR-21, miR-126, miR155, miR-182), and genes related to the adherens junction pathway, including EGFR (miR-21, miR-133a, miR145, miR-146a and miR-155), TGFBR1 (miR-21, miR145, miR-196b). Finally, several genes were involved in the proteoglycans in cancer pathway that regulated MAPK1, FN1, FZD4, CTNNB1, RDX, MSN, SDC2, ACTG1, and IGF1R. The proteoglycans in cancer pathway modulate the dynamics and kinetics of various ligand-receptor interactions that appear to play a role in CAD pathogenesis.

## Discussion

Although enormous progress has been achieved to diagnose and treat CAD with invasive techniques, serious cardiovascular events occur to a large percentage of patients with this disease [22, 23]. These serious events can be partly referred to as unraveled molecular events that lead to CAD pathogenesis, most likely involving atherosclerosis and genetic factors [23–25]. In the search for reliable biomarkers for CAD, circulating miRNAs biostable nature, encouraged research in this area aiming to use it as non-invasive biomarkers [26]. Given a possible clinical transferability of our results, we have isolated circulating miRNAs from EDTA-plasma, for investigating a panel of 14 circulating miRNAs shown in Table 1 relying upon the previously reported results for the sensitivity of the qRT-PCR for the extracted miRNAs from plasma [27, 28].

In accordance with our results, Ren et al., Tsai et al., and Li et al., [12, 29, 30] reported the up-regulation of miR-21 in CAD. This miR-21 up-regulation could be due to the associated effects of vascular wall-shear stress on the endothelium and oxidative stress [31–33] and due to the effect of the oscillatory shear stress that contributes to the vascular endothelium proinflammatory responses. Ren et al., Liu et al., Jansen et al., Wagner et al., and D'Alessandra et al. [12, 34–37] showed upregulation of miR-126 in their CAD research. MiR-126 is responsible for endothelial cell repair and vascular development, and the endothelial cells is enriched with it [38, 39]. Xu et al. showed upregulation of miR-135b among CAD patients compared to controls. MiR-135b targets the MEF2C gene, which is mainly involved in cellular homeostasis, cell proliferation, and migration in the cardiovascular system, which affects the cells phenotype [39–42]. Maciejak et al., Zhu et al., and Choteau et al. [43–45] documented upregulation of miR-145 in CAD. MiR-145 is abundant in vascular smooth muscles. Its expression is dysregulated in atherosclerotic vessels [46]. Niculescu et al. and Dégano et al. [47, 48] reported the upregulation of miR-146a among CAD patients. MiR-146a is implicated in both inflammation and lipid homeostasis [48, 49]. MiR-146a functions by its inhibitory effect on oxidized low-density lipoproteins and inflammatory response [50], thus affecting the pathogenesis of atherosclerosis [33]. Zhu et al. documented in their work the upregulation of miR-182 [51]. Xu et al. reported upregulation of miR-205 resembling our findings. MiR-205 is recently discovered to decrease cellular proliferation, hinders invasion, and increase apoptosis [52, 53]. Liu et al., Schulte et al., and Shan et al. [34, 54, 55] reported upregulation of miR-223, resembling our study results. MiR-223 is thought to regulate endothelial cells inflammation and appears to be associated with HDL [56, 57]. Magenta et al. [58] reported upregulation of miR-200b and highlighted that it is overexpressed in atherosclerosis, ischemic muscles, and vascular dysfunction. Fichtlscherer et al. and Weber et al. [59, 60] reported downregulation of miR-155 as our results. MiR-155 is known to be implicated in inflammatory responses where it strengthen inflammation and sustain macrophages [61, 62]. Finally, Patterson et al. showed downregulation of miR-133a and 208a in CAD patients [63].

On the contrary to our results, D'Alessandra et al., and Liu et al. [37, 64] reported upregulation of miR-133a and miR-208a, which was significantly downregulated in our study. Fichtlscherer et al. [59] reported downregulation of miR-126 and miR-145, while Weber et al., Gao et al., Ying et al., and Wagner et al. [60, 65–67] detected downregulation of miR-145. Ying et al. [66] reported downregulation of miR-196 while Wagner et al. [67] showed downregulation of miR-223. MiR-145 and miR-182 were the most upregulated miRNAs in our study, although miR-145 is not usually upregulated in CAD patients.

From the abovementioned, we deduce that there is no consensus on the relative expression signature of circulating miRNAs in CAD. So, we should interpret miRNA results with caution due to these contradicting results. These contradictions can be explained by which type of body fluid was used prior to miRNA extraction, how the sample was prepared and preserved, which platform was used for the analysis. Going into more sophisticated details, RNA extraction method itself can affect the concentration and quality of miRNAs extracted [68]. Also, the normalization strategy whether mono or multiple endogenous controls were used is important [69–73]. Moreover, during sample collection and preparation step, centrifugation is a necessary procedure for blood. The centrifugation helps in starting with high-quality plasma for miRNA extraction [71]. These methodological variations could lead to conflicting results between different studies. In our opinion, standardization of various methodologies among all studies investigating circulating miRNAs can help in solving the contradicting results problem in the future.

According to ROC analysis results for unraveling the discriminating power of the circulating miRNAs under study, miR-145, miR-182, miR-205, and miR-133a were found to be highly predictive as potential biomarkers for discriminating CAD patients from controls as their AUCs values was above 0.80 [74]. MiR-182 and miR-205 are recently linked miRNAs in cardiovascular disease. Thus, these four circulating miRNAs may be used as a panel for the detecting underlined CAD pathogenesis. However, the sensitivity and specificity of this four circulating miRNAs panel need to be further investigated in a larger cohort.

Considering the relation between miRNAs and the clinical data, most of the studied miRNAs showed a statistically significant relation with age. Only miR-133a and miR-155 showed a significant inverse correlation with age, and this was consistent with the results of Fichtlscherer et al. [75]. In the same consensus, miR-223 was directly correlated with age, as shown by Schulte and his colleagues [54]. On the contrary, Ali et al. [76] showed different results with no correlation between their miRNAs understudy and age. Regarding special habits, very few studies studied the role of smoking with circulating miRNAs in CAD. Our results showed that miR-21, miR-126, miR-135b, miR-155, and miR-182 was significantly correlated with smoking. In contrast to our study results, miR-145 was significantly associated with smoking, as reported by Gao and his colleagues [77]. Although our study reported a significant correlation between miR-133a and family history, another Egyptian study by Turky et al. didn't correlate miR-133a with family history in CAD [78]. All miRNAs under study except miR-208 revealed a statistically significant relation with dyslipidemia. Following our results, ElShafea et al. in Egypt reported a significant reverse correlation with dyslipidemia [79]. Faccini et al. reported upregulation of miR-140 and that its antagonism could be a new therapeutic strategy for treating hypercholesterolemia and atherosclerosis [68]. In contrast, Fujii et al. contradicted our results and reported that miR-126 wasn't correlated with dyslipidemia [80]. Considering obesity, our study and another Egyptian study done by Turky et al. showed that miR-133a showed significance with the obesity in CAD patients [78]. Other miRNAs in our study were not correlated with obesity. In comparison, miR-126 and miR-155 showed significance with BMI grade. Jusic et al. reported the association of miR-21 with hypertension in CAD patients [81], which was not the case in our study, but miR-140b, miR-182, miR-196b, and miR-208 showed to be correlated with hypertension. Regarding ischemic heart disease, Jansen et al. reported that miR-126 correlates with IHD in CAD patients [82], which was not the case also in our study. Instead, miR-21 and miR-145 showed a significant correlation with IHD in CAD patients. Finally, the observed correlation between miR-135b and miR-140b and WMSI was not reported before in the literature. This finding may be attributed to the fact that miR-135b and miR-140b overexpression has a role in blood vessel endothelial cell migration and proliferation, impaired cardiac conduction system activity, enhanced cardiomyocytes apoptosis, and decreased resistance to reactive oxygen species (ROS) that can cause changes in the vessel walls [83, 84].

The combined action of the 14 studied miRNAs revealed number of pathways regulating fatty acid biosynthesis and ECM-receptor interaction (Fig. 4). As the pathways identified in our study have been proved to be associated with CAD [85, 86], we can deduce that this group of miRNAs are mostly implicated in the pathogenesis of CAD with their predicted and/or validated function and biomarker type are summarized in Table 5.

**Table 5:** Selected miRNAs under study involved in CAD pathogenesis with their predictive/ validated function and biomarker type

MiRNA	Predictive/ validated function	Biomarker	References
MiR-21	Share in the proinflammatory processes in the vascular endothelium, Promotes atherosclerosis	Diagnosis Severity	Fleissner et al., Zhou et al. and Weber et al., [31, 33, 60]
MiR-126	Increase EC proliferation, Protects against atherosclerosis	Prognostic Diagnosis Severity	Kuhnert et al., and Urbich et al. [38, 46]
MiR-133a	Promotes myogenesis, Cardiac conductance, Controls collagen synthesis and fibrosis	Diagnosis Prognostic Severity	Ahlin et al., Liu et al. and Laffont et al. [89–91]
MiR-135b	Positive regulation of blood vessel endothelial cell migration, and proliferation	Prognostic Treatment Prediction	Maiti et al., Potthoff et al. and Lin et al. [39, 41, 42]
MiR-140	Negative regulation of NF-kappaB activity, and interleukin-6 production	Diagnosis Prognostic	Werner et al. and Taurino et al. [84, 92]
MiR-145	Increases collagen in the plaque, Increase stability of the plaque, Protects against atherosclerosis	Severity Diagnosis	Cordes et al. and Wei et al. [93, 94]
MiR-146a	Inhibits lipid accumulation, Decrease inflammatory response, Prevents atherosclerosis	Prognostic Severity	Taganov et al. and Yang et al. [95, 96]
MiR-155	Increases inflammation, Increases atherosclerosis	Severity Diagnosis	Nazari-Jahantigh et al., Wei et al. Androulidaki et al., and Du et al. [61, 62, 97, 98]
MiR-182	Affected by angiogenesis causing modulation in the myocardial response	Diagnosis Treatment Prediction	Zhu et al., and Li et al. [51, 99]
MiR-196b	Modulates the cardiomyocyte hypertrophy, Associated with peripheral arterial disease	Prognostic Treatment Prediction	Stather et al. and Wu et al. [100, 101]
MiR-200b	Promotes endothelial cell apoptosis Regulation of myotube differentiation and angiogenesis	Prognostic Treatment Prediction	Zhang et al. [102]
MiR-205	Regulating oxidative stress, mitochondrial function, and apoptosis thus affecting cardiac ischemia/ reperfusion injury	Prognostic Treatment Prediction	Xu et al. [52]
MiR-208a	Has a role in cardiac development, Regulate cardiac myosin heavy chain expression	Severity Diagnosis	Chistiakov et al. [103]
MiR-223	Affects inflammation in endothelial cells, Increases atherosclerosis	Prognostic	Vickers et al. and Tabet et al. [56, 57]

MiRNAs function via modulating the expression of its target messenger RNA, thereby affecting important biological processes [87]. By investigating the potential biological role of miRNA-specific target genes in our study, most of the target genes were enriched in the biological process of negative regulation of transport, regulation of cardiomyocyte differentiation, negative regulation of cytokine production, regulation of muscle cell differentiation, regulation of smooth muscle cell proliferation, miRNA-mediated gene silencing by inhibition of translation and gene silencing by miRNA. These results emphasized that our deregulated miRNAs under study play an important role in CAD and is participating in various signaling pathways that is related to circulatory function. Circulating miRNAs shown to affect target mRNA expression in different cells [88], So, using miRTargetLink 2.0 as shown in (Fig. 6), we predicted the target genes for the miRNAs understudy to understand their biological roles in CAD. We found that the 14 miRNAs understudy may affect several aspects of atherosclerotic plaques, such as inflammation, hypoxia, angiogenesis, inflammation, apoptosis, and ECM degradation (Table 5). They may also regulate several key-signaling pathways in atherosclerotic plaques, such as pathways involving toll-like receptor-4 (TLR-4), hypoxia-inducible factor 1a, and (HIF-1a), transforming growth factor-b (TGF-b), and FOXO signaling pathway.

Concerning our study limitations, this is a mono center study involving a limited number of patients and we investigated only 14 human miRNAs. So, we recommend doing multicentric studies across different geographical locations in the region with larger study populations. Also, we cannot exclude that other miRNAs not investigated in our study is not implicated in CAD pathogenesis. Moreover, several confounders like age, smoking, family history, and dyslipidemia differed between the CAD and control groups. So, the expression of these miRNAs could have been affected by these confounders. Thus further in vitro, in vivo, functional and clinical validation studies could help in better understanding of the precise role of miRNAs in CAD and in validating this study findings.

## Conclusion

In conclusion, the results of this study identified a differentially expressed circulating miRNAs signature that can discriminate CAD patients from control subjects. These results provide new insights into the pivotal role of miRNAs expression associated with CAD pathogenesis. The potential diagnostic value of circulating miRNAs has been shown in CAD patients as depicted through our discussion. Our study results extends these findings and confirm that CAD patients show specific circulating miRNAs signature. Also, we revealed novel findings regarding correlation with clinical data where we reported that miR-133a and miR-182 showed significant relation with family history. MiR-140b, miR-182, miR-196b, and miR-208 revealed a positive statistically significant link with hypertension, while miR-21 and miR-145 showed significance with ischemic heart disease. Finally, miR-135b and miR-140b showed to be correlated with WMSI. However, owing to the current overlap of the signatures pinpointed from various studies, we recommend further studies in relation to the miRNAs discriminating power in CAD. These future studies should preferably standardize the laboratory methodology, address larger population size, implementing functional and clinical validation studies to help better understanding the underlying clinical significance and miRNAs role in CAD development. Finally, we can declare that despite barriers to implementing miRNA-based studies in CAD, our research results foresee it as promising non-invasive biomarkers in CAD. To the best of our knowledge, this is the first study in Egypt to assess a CAD mQ11iRNAs panel encompassing 14 biomarkers.

## Supplementary Information

**Additional file 1.** Study main results including clinicopathological data of the study participants and miRNAs expression values.

**Additional file 2.** Study detailed subjects and methods.

## Data Availability

The datasets used and/or analyzed during the current study are available from the corresponding author on reasonable request.
