# Supplementary material for: Identification of a circulating microRNAs biomarker panel for non-invasive diagnosis of coronary artery disease: case–control study
Source: BMC Cardiovasc Disord. 2022 Jun 24;22:286. doi: 10.1186/s12872-022-02711-9 (PMC9233383; doi:10.1186/s12872-022-02711-9)
Supplement: Supplementary file 2 — Additional file 2. Study detailed subjects and methods. [file 12872_2022_2711_MOESM2_ESM.docx]

**2. Subjects and Methods**

**2.1. Study Population.** This study was a case-control study with 146 participants classified into two groups. The first group included 73 patients presenting with symptoms or findings suggesting CAD by clinical examination and diagnostic tools (Echo and ECG) recruited from the cardiology clinic at the Suez Canal University Hospital (SCUH) from June 2020 till June 2021. Patients aged 18 to 85 years from both genders were enrolled to collect demographic data, family history of CAD, medical history, and lifestyle habits. Patients with other chronic cardiovascular diseases or congenital heart disease were excluded. Risk factors were determined by medical diagnosis and/or treatment for hyperlipidemia, hypertension, and diabetes. Clinical examination was done for all patients under study, including vital signs, general examination, and local cardiac examination. Anthropometrical measurements included height, body weight, waist circumference, and body mass index (BMI) were calculated (kg/m2). Blood pressure and resting heart rate were measured with an automatic sphygmomanometer after ≥5 min rest. Two measurements were performed, and average values were recorded. Echocardiography was done at the Cardiovascular Department of the SCU Hospital to assess the LV size, LV systolic function (ejection fraction), and diastolic dysfunction and was evaluated independently by two operators. Patients with CAD were diagnosed based on at least one of the followings; positive stress imaging (Exercise stress ECG, stress echocardiography, SPECT), presence of wall motion abnormalities in resting echocardiography, prior history of CCU admission with CAD or prior coronary angiography diagnosing obstructive CAD or previous coronary stenting.

The second group included 73 healthy controls from SCUH blood bank from both genders, aged 18 and older, not taking medications or suffering from any cardiovascular or chronic disease. All subjects signed a written informed consent prior to their enrollment.

**2.2. Selection of miRNAs under study using bioinformatics tools.** The miRNAs under study were selected using bioinformatics online tools as HMDD (http://www.cuilab.cn/) [101], and miR2Disease (http://www.mir2disease.org/) [102], and via searching the literature as shown in Table (A).

**Table (A).** Selected miRNAs under study involved in CAD based on literature search

| **MiRNA** | **Predictive/ Validated Function** | **References** |
| --- | --- | --- |
| **MiR-21** | - Share in the proinflammatory processes in the vascular endothelium, - Promotes atherosclerosis | Fleissner et al., Zhou et al. and Weber et al, **[31,33,60]** |
| **MiR-126** | - Increase EC proliferation, - Protects against atherosclerosis | Kuhnert et al., and Urbich et al. **[38,46]** |
| **MiR-133a** | - Promotes myogenesis, - Cardiac conductance, - Controls collagen synthesis and fibrosis | Ahlin et al., Liu et al. and Laffont et al. **[88-90]** |
| **MiR-135b** | - Positive regulation of blood vessel endothelial cell migration, and proliferation | Maiti et al., Potthoff et al. and Lin et al. **[39,41,42]** |
| **MiR-140** | - Negative regulation of NF-kappaB activity, and interleukin-6 production | Werner et al. and Taurino et al **[85,91]** |
| **MiR-145** | - Increases collagen in the plaque, - Increase stability of the plaque, - Protects against atherosclerosis | Cordes et al. and Wei et al **[92,93]** |
| **MiR-146a** | - Inhibits lipid accumulation, - Decrease inflammatory response, - Prevents atherosclerosis | Taganov et al. and Yang et al **[94,95]** |
| **MiR-155** | - Increases inflammation, - Increases atherosclerosis | Nazari-Jahantigh et al., Wei et al Androulidaki et al., and Du et al. **[61,62,96,97]** |
| **MiR-182** | - Affected by angiogenesis causing modulation in the myocardial response | Zhu et al., and Li et al. **[51, 98]** |
| **MiR-196b** | - Modulates the cardiomyocyte hypertrophy, - Associated with peripheral arterial disease | Stather et al. and Wu et al. **[99,100]** |
| **MiR-200b** | - Promotes endothelial cell apoptosis - Regulation of myotube differentiation and angiogenesis | Zhang et al. **[101]** |
| **MiR-205** | - Regulating oxidative stress, mitochondrial function, and apoptosis thus affecting cardiac ischemia/ reperfusion injury | Xu et al. **[52]** |
| **MiR-208a** | - Has a role in cardiac development, - Regulate cardiac myosin heavy chain expression | Chistiakov et al. **[102]** |
| **MiR-223** | - Affects inflammation in endothelial cells, - Increases atherosclerosis | Vickers et al. and Tabet et al. **[56,57]** |

**2.3. Blood Samples Collection.** Three ml of fresh venous blood was collected from all study participants in vacutainer tubes containing ethylene diamine tetraacetic acid (EDTA) anticoagulant. The samples were centrifuged to separate plasma; 100 μl plasma was preserved in 500 μl Qiazole reagent. The plasma samples were stored at -80℃ till further analysis.

**2.4. MicroRNA extraction and quality analysis.** Total RNA was isolated using Qiagen miRNeasy Mini kit (cat no 217004, QIAGEN, Hilden, Germany) following the modified protocol supplied by the manufacturer. RNA concentration and purity were determined using NanoDrop 2000 1C spectrophotometer (NanoDrop Tech., Inc. Wilmington, DE, USA).

**2.5. Circulating miRNAs relative expression analysis using quantitative real-time PCR assay.** The expression profile of 14 circulating miRNAs involved in CAD pathogenesis was assessed in the plasma of all study participants using Real Time-Polymerase Chain Reaction (RT-PCR). This was done via a two-step approach as follows; (a) reverse transcription (RT), where complementary DNA (cDNA) was generated from total RNA containing miRNA using the miScript II RT Kit (Qiagen, Catalog no. 218161), in which miRNAs and other noncoding RNAs (ncRNAs) were polyadenylated by poly(A) polymerase and converted into cDNA by reverse transcriptase with oligo-dT priming. RT was carried out in Veriti™ 96-Well Thermal Cycler (Applied Biosystems, USA) at 37 °C for 1 hour, followed by inactivation of the reaction by briefly incubating at 95 °C. (b) quantitative Real-Time PCR, where the premix of cDNA was used as a template for relative quantification of the 14 human miRNAs under study, which are miR-21-3p, miR-126-5p, miR-145-5p, miR-155-3p, miR-208a-5p, miR-140-3p, miR-182-5p, miR-146a-5p, miR-223-5p, miR-196b-5p, miR-200b-3p, miR-205-5p, miR-133a-5p, and miR-135b-5p using miScript SYBR Green PCR Kit (Qiagen, cat. no 218076) with a universal reverse primer and a specific forward primer sequence for each miRNA as presented in Table 6. The expression levels were done according to the minimum information for publication of quantitative RT- PCR experiments (MIQE) guidelines. The reactions were run in duplicates with a "No-RT", and a "No-template" controls were included in each run. Each PCR run started at 95°C initially for 5 min, followed by 40 cycles of denaturation, annealing and elongation at 95°C (15 s), 55°C (1 min), and 72°C (1 min) respectively. The SNORD68 and RNU6B average was used as endogenous control to enable data analysis using the ΔΔCT method of relative quantification.

**Table (B).** Primer Sequences of miRNAs under study

| **MiRNA** | **Primer Sequence** |
| --- | --- |
| **MiR-21-3p** | GCTTATCAGACTGATGTTG |
| **MiR-126-5p** | CATTATTACTTTTGGTACGC |
| **MiR-133a-5p** | TTTGGTCCCCTTCAACCAGC |
| **MiR-135b-5p** | GGCTTTTCATTCCTATGTG |
| **MiR-140-3p** | CAGTGGTTTTACCCTATG |
| **MiR-145-5p** | GTCCAGTTTTCCCAGGA |
| **MiR-146a-5p** | GAGAACTGAATTCCATGG |
| **MiR-155-3p** | TGCTAATCGTGATAGGGG |
| **MiR-182-5p** | GGCAATGGTAGAACTCAC |
| **MiR-196b-5p** | GGTAGTTTCCTGTTGTTG |
| **MiR-200b-5p** | CTTACTGGGCAGCATTG |
| **MiR-205-5p** | TCCTTCATTCCACCGGA |
| **MiR-208a-5p** | ATAAGACGAGCAAAAAGCT |
| **MiR-223-5p** | CGTGTATTTGACAAGCTG |
| **SNORD68** | GCCCCTGCGCAAGGATGAC |
| **RNU6B** | GCCCCTGCGCAAGGATGAC |

**2.6. Assessment of circulating miRNAs predictive significance as biomarkers.** The contribution to the predictive capacity of the significant miRNAs was analyzed using Receiver Operating Characteristic (ROC) curves to evaluate the diagnostic value of the used miRNAs as biomarkers for CAD pathogenesis. A p-value of <0.05 was considered statistically significant.

**2.7. Function and pathway enrichment analysis.** The functional enrichment analysis was conducted using the software Database for Annotation Visualization and Integrated Discovery (DAVID) (https://david.ncifcrf.gov/) [107], where gene ontology (GO) consisting of biological processes, cellular components, and molecular functions terms was searched for via Pathway analysis on the Kyoto Encyclopedia of Genes and Genomes (KEGG) database [108] for determining the pathways affected with differential miRNA expression and their target genes. In detail, sorting out through hypergeometric and Fisher tests was done for enriched biological processes, cellular components, molecular functions, and signaling pathways after mapping the potential target genes to the dataset of GO annotations and KEGG pathways. False discovery rate (FDR) adjustment was performed to judge the significance of differences in multiple testing, and a q<0.05 was considered to indicate a statistically significant result. Also, using the metascape tool (http://metascape.org/) [109], analysis had been carried out for each miRNA understudy, pathway, and process enrichment with the following ontology sources: Cell Type Signatures, DisGeNET, PaGenBase, KEGG Pathway, GO Biological Processes, Transcription Factor Targets, and WikiPathways. All genes in the genome had been used as the enrichment background. Terms with a p-value < 0.01, a minimum count of 3, and an enrichment factor > 1.5 (the enrichment factor is the ratio between the observed counts and the counts expected by chance) were collected and grouped into clusters based on their membership similarities. More specifically, p-values were calculated based on the accumulative hypergeometric distribution, and q-values were computed using the Banjamini-Hochberg procedure for accounting for multiple testings. Kappa scores were used as the similarity metric when performing hierarchical clustering on the enriched terms, and sub-trees with a similarity of > 0.3 were considered a cluster. The most statistically significant term within a cluster was chosen to represent the cluster. Finally, quality control and association analysis were identified for our studied miRNAs list enrichments in the following ontology categories: DisGeNET. All genes in the genome had been used as the enrichment background. Terms with a p-value < 0.01, a minimum count of 3, and an enrichment factor > 1.5 (the enrichment factor is the ratio between the observed counts and the counts expected by chance) were collected and grouped into clusters based on their membership similarities. The top few enriched clusters (one term per cluster). The algorithm used was the same pathway and process enrichment analysis.

**2.8. MiRNA‐mRNA regulatory network construction**. The targets of the homogenously statistically significant DEmiRNAs were predicted using miRTargetLink 2.0 (Version 2.0, https://ccb-compute.cs.uni-saarland.de/) [110]. Homogenously statistically significant DEGs targets were retained, and the negative correlation of the miRNA- mRNA pairs were included. Finally, the gene modules targets were highlighted, followed by visualization of the screened miRNA-mRNA pairs.

**2.9. Statistical Analysis.** Data were analyzed using R software version 3.3.2, GraphPad prism 7, SPSS software version 23.0, and PC-ORD ver. 5.0. We used the G*Power 3.1.9.2. with the specified study design (gene expression), alpha error = 0.05, an effect size = 0.74, and a total sample size of 146 was calculated that can give 80% power of the study http://www.gpower.hhu.de/A [111]. Fold change of the miRNAs was estimated using the LIVAC method (=2-ΔΔCq) [112]. Quantitative data were expressed as median and quartiles or means ± standard deviation, while qualitative data were expressed as numbers and percentages. Data distribution and variance homogeneity were checked by Levene's tests and Shapiro-Wilk. Student-t, Two-sided Chi-square, ANOVA, Kruskal-Wallis, and Mann-Whitney U tests were used as appropriate. Correlation analysis by Spearman's rank test was carried out.
